# LunAero: Automated “smart” hardware for recording video of nocturnal migration

**DOI:** 10.1016/j.ohx.2020.e00106

**Published:** 2020-03-18

**Authors:** Wesley T. Honeycutt, Alyse V. Heaston, Jeffrey F. Kelly, Eli S. Bridge

**Affiliations:** Oklahoma Biological Survey, University of Oklahoma, 111 Chesapeake St., Norman, OK 73019 USA

**Keywords:** Nocturnal, Migration, Ornithology, Research techniques, Aeroecology, Moon, Hardware

## Abstract

Moon watching is a method of quantifying nocturnal bird migration by focusing a telescope on the moon and recording observations of flying birds silhouetted against the lunar surface. Although simple and well-established, researchers use moon watching infrequently due in part to the hours of late night observation it requires. To reduce the labor entailed in moon watching, we designed a low-cost system called LunAero that can track and record video of the moon at night. Here we present a proof-of-concept prototype that can serve as a platform for citizen scientists interested in observing nocturnal bird migration. We tested the video recording on clear nights from February 2018 to May 2019 when the moon was full or nearly full. Manual analysis of a 1.5 h sample of video revealed a total of 450 birds, which is a much higher detection rate than previous moon watching efforts have yielded. The hardware described here is part of a larger effort involving software development (currently underway) to automate recorded video analysis. We argue that LunAero can reduce the labor involved in moon watching, offer improved data quality over traditional moon watching, and provide insights into social behavior and wind-drift compensation in migrating birds.

## Graphical abstract

**Figure:**
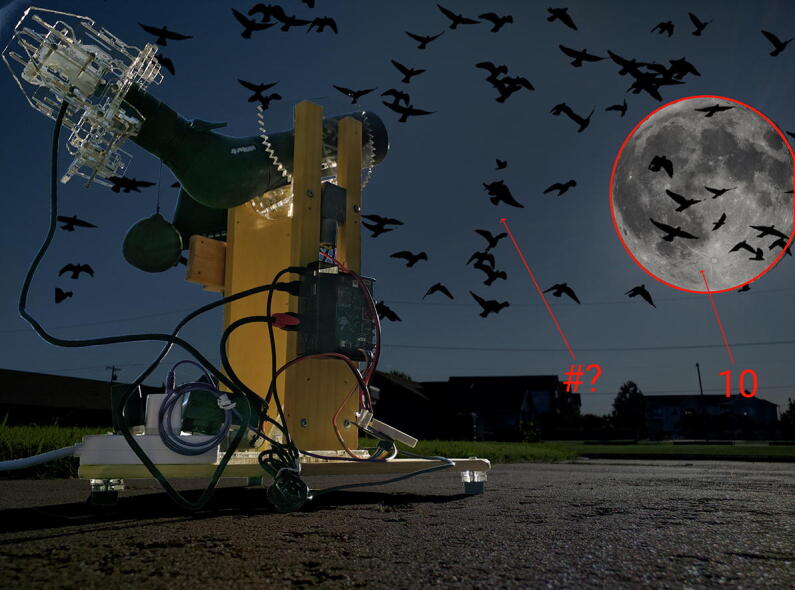

**Specifications table:**

**Table:** 

Hardware name	LunAero
Subject area	Biological Sciences
Hardware type	Field measurements and sensors
Open source license	MIT License
Cost of hardware	<$400
Source file repository	https://doi.org/10.17605/OSF.IO/N7ZEK

## 1 Hardware in context

Bird migration is both a fascinating and ecologically important phenomenon that influences many natural communities by transfer of biomass [1], [2] over large distances and providing vectors for diseases and parasites [3]. However, our ability to observe and quantify bird migration is limited by the fact that most avian migrants are small (<30 g see [4]), and they generally make their migratory flights at night. Remote sensing techniques such as radar and audio monitoring allow for quantification of bird migration [5], [6], [7], but there are few options available for making direct observation of nocturnal migrants.

One venerable and well-tested means of observing migration is to simply focus a telescope on the moon and use it as a “backdrop” to note the passage of individual birds overhead. This observational method, known as moon watching, was first described by W. E. D. Scott, in 1881 following a fortuitous observation of flying birds during an astronomy demonstration [8]. Following Scott’s description, ornithological moon watching received only modest attention until George H. Lowrey and colleagues honed the technique, publishing a comprehensive guide to moonwatching in 1951 [9] followed by a nationwide collaborative moon-watching effort [10]. Lowery established protocols for determining flight direction and estimation of nightly passage rates, which were expanded upon by contemporaries [11]. However, since Lowrey’s efforts, moon watching has had only limited application (see [12], [13], [14], [15]), has often been employed only for casual observations (*e.g.* [16]), or used secondary to radar or infrared methods (*e.g.* [17], [18]). To some extent, the use of radar superseded moon watching as a means of observing and quantifying nocturnal bird migration [19], [6]. In 1995 Liechti and Bruderer [20] published a side-by-side comparison of moon watching, thin-beam radar, and thermal imaging, which revealed that moon watching was likely to underestimate bird passage rates due to human error (*i.e.* missed observations). Moreover, moon watching requires a considerable investment of labor and, for diurnal researchers, lost sleep. However, as noted by Liechti and Bruderer [20], moon watching is cost-effective technique which can be deployed on a large scale, whereas high-quality thermal cameras and radars capable of identifying individual birds are not widely available. We argue that if the labor and inconvenience associated with moon watching could be diminished, along with the potential for human error, then moon watching could become a widespread and important tool for migration monitoring in areas with frequent clear skies, potentially engaging citizen-scientists as its primary practitioners.

In an effort to streamline the moon watching method, we developed a system that automates the process through use of a CCD camera, a small computer, a spotting scope, a motorized mount, and some simple computer vision techniques. The system, dubbed LunAero, performs the relatively simple task of recording high-definition video with sufficient magnification to observe and quantify nocturnal bird migration. The hardware described in this paper is part of a larger effort that involves development of software for analyzing the recorded video. This video analysis software is to be the subject of another publication. In this paper we describe a working LunAero system and present the results of testing performed during peak bird migration in the spring of 2018 along with some preliminary analyses of bird densities and flight behavior.

## 2 Hardware description

**Fig. 1:**
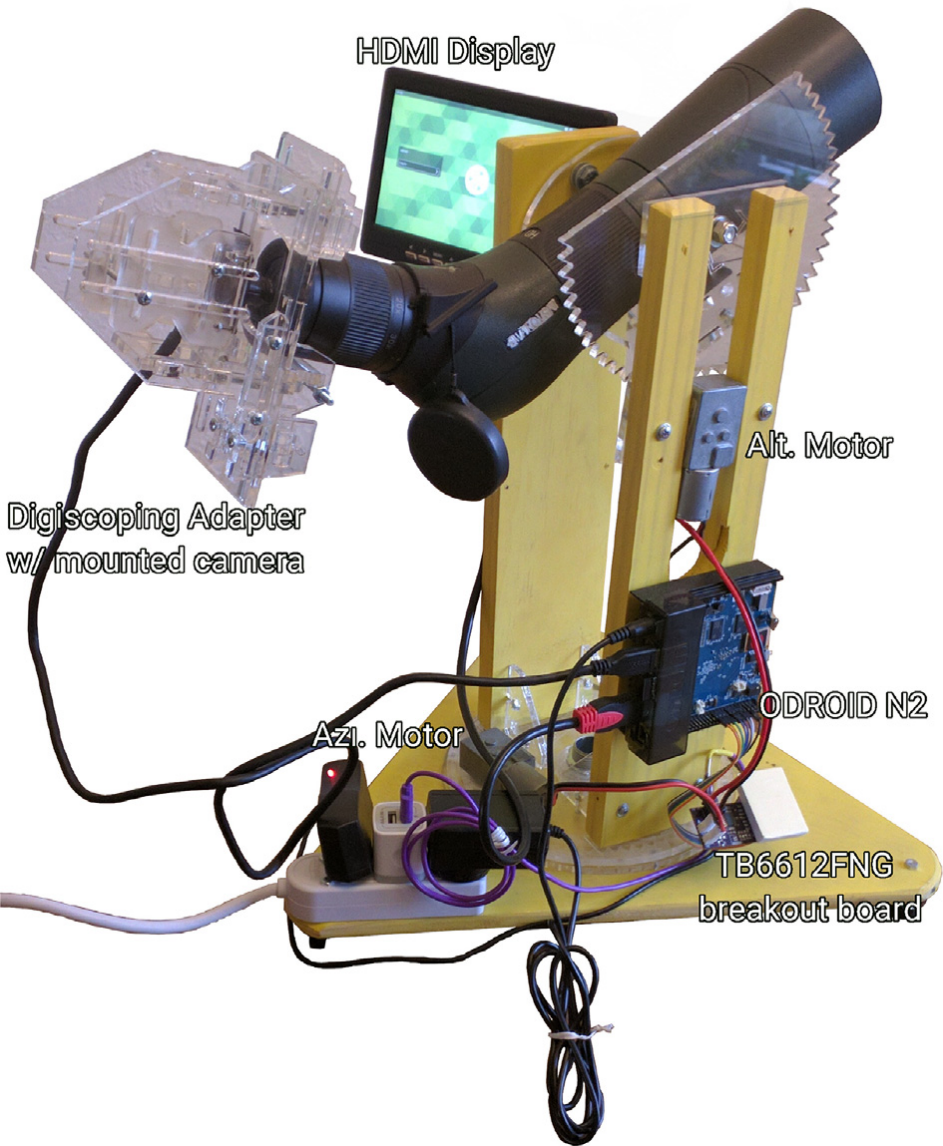
Photograph of an assembled LunAero unit.

The LunAero system consists of a motorized, two-axis mount for a spotting scope or a telescope that has been integrated with a CCD camera via a small computer. The CCD camera is mounted to the scope such that it can record magnified images of the moon allowing the computer to both store video footage and adjust the position of the scope as needed via the motorized scope mount (see Fig. 1). In designing LunAero we sought a device that could reliably produce high-quality video footage while remaining inexpensive and accessible to amateur scientists. Astronomers typically view celestial objects using ephemeral models [21] and motorized tracking systems capable of continuous, precise motion. We found this approach to be impossible to emulate using low-cost components. Hence, we opted for a machine-vision feedback system, wherein a simple motorized telescope mount was controlled in a manner that maintained the moon within the field of view.

With this design strategy, we were able to produce LunAero from readily sourced parts and materials that are easily obtainable by research groups and citizen scientists with minimal technical experience. Detailed design files and guidelines for construction are online at Open Science Framework repository for collected data at https://doi.org/10.17605/OSF.IO/N7ZEK.

### 2.1 Mechanical parts

The core mechanical portion of the automated moon tracking device is an altitude-azimuth mount that supports a wide array of telescopes. Spotting scopes in particular are a pervasive tool of amateur bird watchers, meaning that many potential users already have one. The scope mounts to a cradle with a 1/4 inch bolt, the standard for tripod mount plates [22], and this cradle interfaces with a DC powered gear motor to allow for vertical movement of the scope. A second gear motor rotates the mount around the vertical axis providing a means of horizontal tracking. The 5 V motors chosen for LunAero provide 3.4 Nm of torque at a speed of about 0.5 rotation/min, and gears associated with the scope cradle and turret further reduce the gear ratio to slow the motion of the scope. A TB6612FNG breakout board capable of supplying 1 A of current drives both motors. A low-cost video camera (described below) mounts to the scope eyepiece via custom digiscoping hardware designed to work with a wide variety of spotting scopes and telescopes. All structural parts are produced from acrylic plastic (polymethylmethacrylate), plywood, and acetal plastic (polyoxymethylene) using a laser cutter and standard woodworking tools. A complete set of designs and circuits is stored at the OSF repository provided above.

### 2.2 Computer control and camera

A key element of the development of LunAero was finding a low-cost computer that could handle computer vision, motor control, and video recording. Prototype versions of LunAero used a Raspberry Pi 3 (Raspberry Pi Foundation, Cambridge, United Kingdom) computer running a distribution of Raspbian Linux. However, we found that the combined demands of image processing and high-resolution video recording were too great for the Raspberry Pi. Therefore, we upgraded to an ODROID N2 (Hardkernel co Ltd., Manan-gu, Anyang, Gyeonggi-do South Korea) to operate the hardware. The N2 is a Unix capable machine with a powerful processor and USB 3.0 ports as well as a general purpose input/output (GPIO) capable of interfacing with the TB6612FNG.

LunAero used a ELP-SUSB1080P01 camera module (Ailipu Technology Co., Ltd, Shenzhen, Guangdong, China) which is based on the Sony IMX291 sensor. The camera module was equipped with a 18 mm fixed focus “S-mount” lens to produce f/2.7 optical characteristics. This configuration produced optics that match well with the target “eye relief” or apparent exit image diameter from the ocular lens of a spotting scope.

### 2.3 Tracking the moon

The moon tracking program works by determining the position of the moon within the camera frame and occasionally activating the motors on the scope mount to bring the moon image close to the center of the field of view. To minimize shakiness in the recorded video, the scope mount remained motionless most of the time, allowing the moon to drift through the field of view. Under this scenario, the mount only needed to move at intervals of approximately 1 m.

The moon tracking program was written for Python 3 [23]. When first executed, it prompts the user to verify the correct date and time; it then allows the user to assume control of the motorized mount via keyboard commands. The user must then use these controls to move the motorized mount until a clear image of the moon is obtained by the camera. The user must then adjust the image exposure as needed and then initiate video recording.

When recording initiated, the program assumes control of the motors and begins the process of maintaining the moon in the camera’s field of view. This tracking process is accomplished by extracting a low resolution frame (800 P  × 600 P) from the camera’s video stream every few seconds and converting the pixels to value arrays using the Numpy [24] and Pillow packages [25]. The program employs a threshold function to distinguish the moon from the background. In the case of fog or mist obscuring the moon, the user may alter this threshold to keep the moon visible to the computer during recording without effecting the recorded video. The Numpy package then determines the centroid of the moon profile to index the moon’s position in the video frame. If this centroid is found too far from a reference point near the center of the frame, then the program activates the motors to correct the position of the moon image in the video frame. Motor control is effected by a software pulse-width modulator that sends signals to the TB6612FNG motor controller using the WiringPi Python package [26]. In cases where clouds obscure the moon, tracking will fail until the cloud passes. Upon complete “loss” of the moon, the program will issue an error and close.

While the robotics keep the moon in view, LunAero uses OpenCV [27] to record video with MJPEG encoding on the eMMC storage drive of the ODROID N2. The system also generates a log file with information on recording time, the number of frames captured, and camera sensor settings. Since Python is an interpreted language, timing is imprecise, necessitating recording clock time and frame counts. This means that although we record video with certain target values such as 30 frames/s, it is likely inconsistent. For time-dependent calculations, such as determining the exact position of the moon as a reference for flight direction calculations, the time value for each frame must be calculated from timestamps in the output log, not the apparent frame rate in the video.

The hardware reported in this publication benefits researchers by:

• Making it easier for researchers to observe nocturnal migration via automatic video recording.
• Increasing the quality of data produced by nocturnal migration observation by allowing researchers to replay video and refine observations.
• Increasing the number of observers at a given site or sites; low-cost hardware is cheaper than hiring technicians.
• Retaining accessibility; hardware costs are minimized such that the technology is accessible to most research groups and enthusiasts.
• Allowing future researchers to apply machine learning and computer vision technology to archived video.

## 3 Design files summary

### 3.1 2D CAD files

The entries in Table 1 list the CAD vector drawings required for fabrication and assembly on 2D CNC equipment. All files are formatted as the commonly used, interoperable Autocad Drawing Interchange Format (DXF). Most modern 2D CNC devices accept vector files in DXF format as direct imports, meaning that these files require little or no CNC or CAD experience to fabricate. The file MoonWatcher_0.225in.dxf is a two-layer vector drawing depicting LunAero parts which a user may cut from standard sheets of 6.35 mm thick acrylic. The primary layer in the drawing represents cut lines while the secondary layer represents etch marks for labels. The file LunAero_acetal_gear_metric.dxf is a single layer drawing for the production of gears from 3.175 mm thick acetal. While these gears can be made from the same acrylic as the majority of the parts in LunAero, acetal gears will produce a smoother video result and will be less prone to fracture.

**Table 1:** CAD Files Manifest.

Design filename	File type	Open source license	Location of the file	
MoonWatcher_0.225in	.dxf	CERN-OHL-1.2	./2D CAD	
MoonWatcher_wood1inch	.dxf			
LunAero_acetal_gear_metric	.dxf

**Table 2:** Software File Manifest.

Design filename	File type	Open source license	Location of the file
__init__	.py	GPL-3.0	./Moontracker_Classes
rpt_control	.py		
__init__	.py		./
ODROID_INSTALL	.sh		
odroid_moontracker	.desktop		
original_moontracker	.py

**Table 3:** Bill of Materials.

Designator	Component	Number	Cost per unit	Total cost	Source of materials
ODROID N2	Computer	1	$84.50	$84.50	Hardkernel Co., Ltd.
eMMC	128 GB drive	1	$65.90	$65.90	Hardkernel Co., Ltd.
TB6612FNG	Motor Controller	1	$4.95	$4.95	Sparkfun Electronics
ELP-SUSB108P01 USB Camera	USB Camera	1	$61.60	$61.60	Alipu Technology Co., Ltd.
5 V 0.5RPM gear motor	Gear Motor	2	$34.98	$69.96	UXCell
Polymer sheets and bolts	Hardware/Materials		$35.00	$35.00	various
Wireless keyboard/touchpad	Keyboard/touchpad	1	$17.99	$17.99	Amazon.com
LCD Display	$5^{\prime \prime }$ 800 × 480 LCD	1	$64.99	$64.99	Amazon.com

The file MoonWatcher_wood1inch.dxf is a single layer drawing to act as a guide for preparing parts from 19.05 mm thick wood, not suitable for direct upload to a cutting instrument. Instead, it should be printed at exact scale and used as a drilling/cutting guide. This design adapts to a user’s needs because the hole spacings and cut lines represent the smallest recommended parts. Therefore, the user may customize the parts, *e.g.* a user with a large scope would need to produce large support arms.

### 3.2 Software files

A file manifest for operation is included in Table 2. The file original_moontracker.py is the main program which runs the LunAero tracking program. Details of the operation of this program are described the Operational Instructions section. The primary program depends on the file rpt_control.py and each __init__ to be present in the directory hierarchy as delivered. The file rpt_control.py is a library for controlling the motors connected to the GPIO ports by the main program. Each __init__ file is a Python library directory file ensuring that the main program has no issues finding its dependencies.

Files ODROID_INSTALL.sh and odroid_moontracker.desktop are present for the convenience of the user. The install script can be executed by the user in Linux to automatically fetch and install dependencies required for the successful operation of the device. The desktop file can be copied to the user’s desktop on the ODROID computer to activate the Python script.

## 4 Bill of materials

A more complete bill of materials including the quantity of individual nuts and bolts required for the project is present on the OSF repository in the Build Instructions subdirectory (see Table 3).

## 5 Build instructions

The LunAero hardware design incorporates a number of acrylic, acetal, and wooden parts, found in the 2D CAD files. Cut all acrylic and acetal parts from sheet stock using the CNC method of choice, following all instrumentation guidelines for that equipment. The plastic parts used in the unit described in this paper were fabricated on an 80 W CO_2_ laser. While amateur ornithologists are unlikely to own personal CNC equipment, access to inexpensive cutting sources has become increasingly available. A survey from [28] in 2015 reports that 26% of “Makerspaces” own and operate a laser cutter CNC for public use [28]. If the user does not have access to a local Makerspace, online vendors offering laser cutting service will cut the parts for a nominal fee. Cut and drill all wooden parts with normal workshop tools, using the 2D CAD file as a printed template for cut and drill locations. Some bolt holes in the acrylic parts require countersinking, and etched circles around the hole denote approximate countersunk perimeter. Each acrylic part in LunAero (excluding a single washer requiring a smooth surface) has been tagged with an etched letter. Use these letter designations as part guides during construction.

**Fig. 2:**
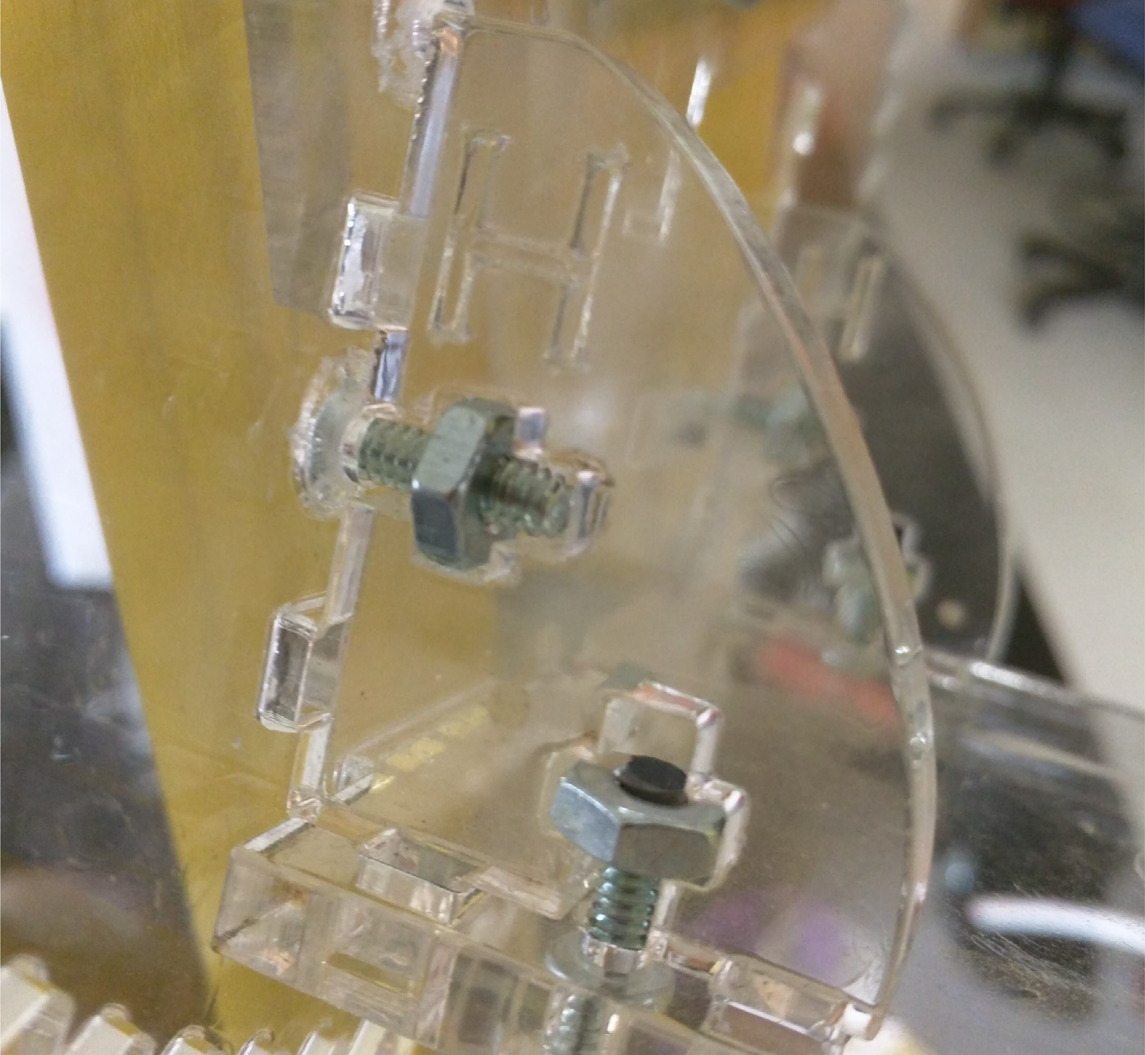
The cruciform cuts on the acrylic parts use a bolt and nut for angled joinery. The pressure of the nut on the cross-piece when the bolt is tightened holds the part in place. In this image, part H is connect to two other parts by cruciform connection.

**Fig. 3:**
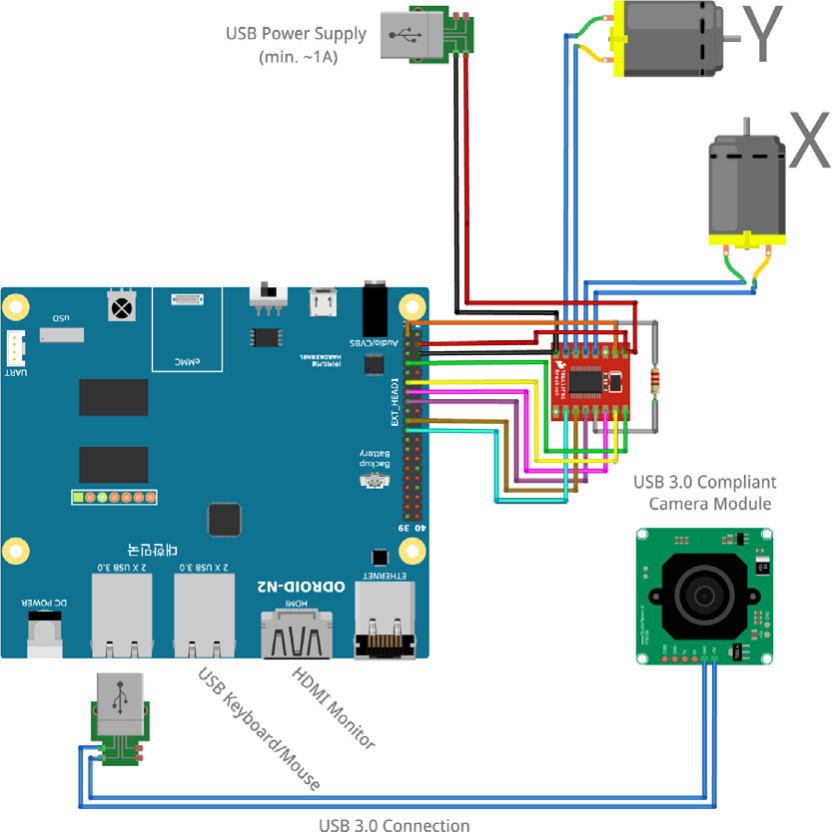
This image illustrates the wiring of LunAero using the ODROID N2 SBC. The parts depicted are all commercially available and may be assembled with minimal electronics experience.

Using the bolts listed on the detailed bill of materials, assemble the LunAero unit. Pictorial instructions for each step of the assembly are available at the OSF repository associated with this manuscript. Note that most of the angled connections in LunAero between flat materials use a “cruciform” bolt and nut connection, shown in Fig. 2. Gears press-fit to the motor axles. The azimuthal motor axle cannot accommodate a retaining collar. Sufficient friction holds gears in place during normal use, if they begin to slip, they require epoxy to hold them in place. Not depicted in the build instructions, the ODROID computer, TB6612FNG board, power supply, and monitor optionally may be affixed to the support arms of LunAero by the user. Fig. 3 depicts the wiring of these electronic parts.

When deploying LunAero, the user’s spotting scope is bolted to one of the drill holes in the cradle with a $1/4^{\prime \prime }$ bolt, per the standard tripod mounting hole on the scope. The user must choose an appropriate hole in the cradle to affix the scope, bearing in mind the center of gravity of the optics. The camera clamp attaches to the eyepiece of the scope. Once attached, the user adjusts the location of the camera within the acrylic housing. Viewing the camera output on the screen, the user tightens the wingnuts to the bolts once the camera shows a complete view of the objective image, free of black edges or distortion. With the camera and scope attached, the LunAero unit is ready to record video.

## 6 Operation instructions

To use the LunAero hardware, the appropriate software must be installed from the OSF repository. In the Linux terminal of a functional ODROID computer running Ubuntu MATE, use wget to pull the software files from the online repository. In this description, we assume that the user has downloaded the software from the repository to a file directory such that the directory tree appears similar to:

If your download appears different, you may experience problems with the next steps. Use the terminal to run the script at /home/%USER%/Documents/LunAero/ODROID_INSTALL.sh as a super user. This script installs all of the prerequisite files and programs required to run the LunAero tracking program. Copy the file /home/%USER%Documents/LunAero/odroid_moontracker.desktop to the desktop of your Ubuntu MATE installation. This desktop file makes it easy to activate the program without using the terminal.

As explained in detail in the instructions included in the OSF repository, the user operates LunAero by acquiring a good-quality image of the moon through the camera, then activating the automatic mode. Placing the LunAero hardware on a flat surface, activate the ODROID computer. Open the moon tracking program by clicking on the desktop icon. When the program starts, go through the time-correction queries, and begin manual tracking. Use the arrow keys on the keyboard to move the scope with the motors until the screen shows the moon completely in view. Adjust the scope magnification and focus until the moon appears crisp to the computer view. Change the brightness of the output image by adjusting the digital camera exposure and ISO in the moon tracking program. The image on the screen should be bright, have sharply focused edges, and the craters of the must be equally crisp to the edge of the moon. Once you are happy with the image quality, press r to quit manual tracking mode and enter automatic mode. At this point computer vision intelligence of the robotics take over and track the bright center of the moon. Barring weather changes or extreme events, LunAero may be left alone to record the moon now. Once you done recording, transfer the video found on the hard drive of the ODROID computer to the storage medium of your choice for archival purposes.

## 7 Validation and characterization

We evaluated the hardware by field testing on nights with clear skies and a moon that was half full or more. We conducted tests in April and May of 2018 and 2019, which roughly corresponded to peak bird migration season. Tests were conducted in Norman, OK, USA, using either a 40× ATS 65 spotting scope (Swarovski Optik, Absam, Austria) or 20–60×80 spotting scope (Alpen Optics). From approximately >70 h of footage collected, we selected a recording sample of 1.5 h beginning at 9:00 PM CDT on April 28, 2018 for manual annotation and analysis. Video annotation was performed by A.H. by playing the video on a desktop computer and noting the time and number of silhouettes present. During annotation, video playback was paused, reversed, and incremented frame-wise to allow for the most accurate object count and timing. This process took approximately 11.5 h to complete.

In addition to this simple annotation we selected an even smaller sample of the same video (9:30 PM–10:30 PM CDT) to manually determine flight directions by measuring transit paths on a computer screen with a protractor. These measurements allowed us to emulate the moon watching protocol developed by Lowrey [9]. In some cases we were also able to discern the head-to-tail orientation of individual silhouettes such that we could compare flight direction and orientation to infer the degree to which orthogonal or oblique winds were affecting particular birds (see Fig. 6).

**Fig. 4:**
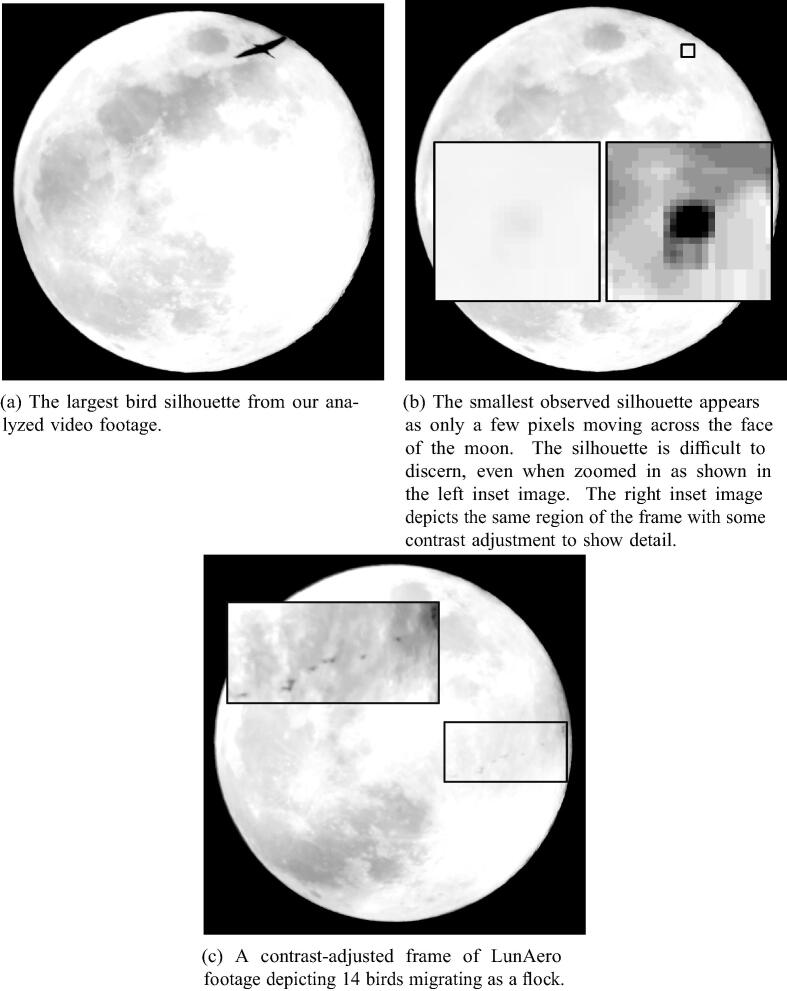
Individual frames extracted from the recorded video show interesting extremes of the application. a and b demonstrate the largest and smallest birds observed by LunAero, respectively. (a) The largest bird silhouette from our analyzed video footage. (b) The smallest observed silhouette appears as only a few pixels moving across the face of the moon. The silhouette is difficult to discern, even when zoomed as shown in the left insert image. The right insert image depicts the same region of the frame with some contrast adjustmenttoshow detail. (c) A contrast- adjusted frame of LUNAero footage depicting 14 birds migrating as a flock.

We counted 450 birds in the 1.5 h test recording, as well as one silhouette that was clearly an insect. The silhouettes ranged greatly in size, with the smallest silhouette scarcely larger than a few pixels (see Fig. 4). Similarly, observation duration for individual birds ranged from 2.14 s to a single video frame .04 s.

**Fig. 5:**
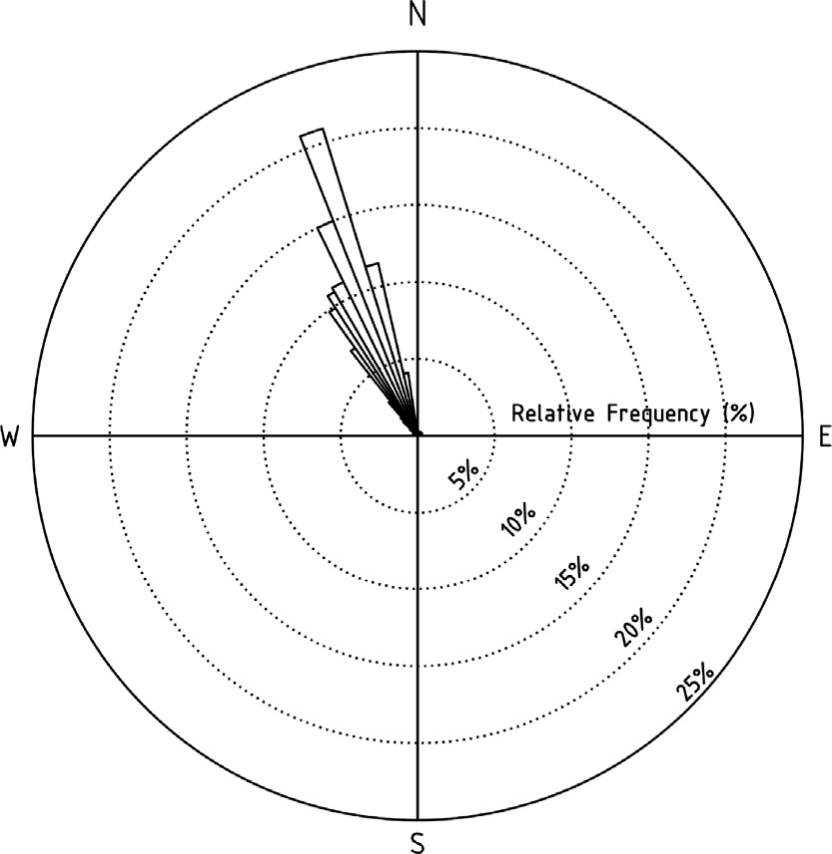
Normalized results of Lowery analysis show a predominant NW path traveled by birds in the analyzed video. This figure depicts the relative frequency of bird count in relation to cardinal direction to the total number of birds recorded during the studied hour of footage.

Analysis of flight directions revealed NW-trending net migration density, reckoned 110° north of east (see Fig. 5). The net traffic rate calculated from the LunAero data was 14,193 birds/kmh (26,289 birds/mh using units matching early nocturnal bird counting papers). This number was derived from Lowery’s moon watching methodology and estimates the number of birds that would fly across a one-mile line on the earth surface per hour [9]. However, we note that Lowery considers this traffic rate to be an arbitrary rate of passage rather than a true density estimate (see Lowery’s text for more detail). What is interesting about our traffic rate estimate is that it exceeds any hourly traffic rate reported in Lowery’s extensive moon watching efforts. This count exceeds even the total seasonal density for more than half of the regions reported from the spring of 1948 from [9]. Although we cannot rule out the possibility that we happened to sample an extraordinary migration event, the most likely explanation for our high traffic rate is that video recording and methodical analysis allowed us to detect more birds than a human can typically discern while doing moon watching in real time. If this explanation is correct, then LunAero has potential to provide much improved migration data compared to traditional moon watching. For positive determination of the cause of the high traffic rate, results from LunAero may be compared with concurrent observations such as radar, however an in-depth comparison of LunAero recordings with radar is not complete at this time.

### 7.1 Lunar studies in the Modern Era

LunAero was first conceived as a means of reducing both the labor required for moon watching and the inconvenience of nighttime data collection. However, our efforts thus far have demonstrated that LunAero surpasses the capabilities of traditional moon watching by enabling improvements in terms of the quality and types of data made available. The most obvious advancement is that LunAero remedies the problem of human error. The ability to detect birds is greatly enhanced in a frame-by-frame examination of recorded video compared to real-time bird counts. In particular, analyses performed on video footage allow for detection of birds that merely skirt the edges of the moon, passing through the lunar backdrop too quickly for most observers to discern in real time. Nisbet et al. lamented these near-edge birds as unusable data [12], but LunAero captures them reliably. Video analysis can be further improved (and made less labor intensive) by using computer vision and machine learning techniques to automatically extract data of interest from recorded moon footage.

In addition to offering improvements in data quality, LunAero footage also allows us to extract new forms of data from a moon watching effort. With video analysis, the relative timing of bird observations can be determined with high accuracy, allowing for quantitative studies of flocking behavior (see Fig. 4c). .

**Fig. 6:**
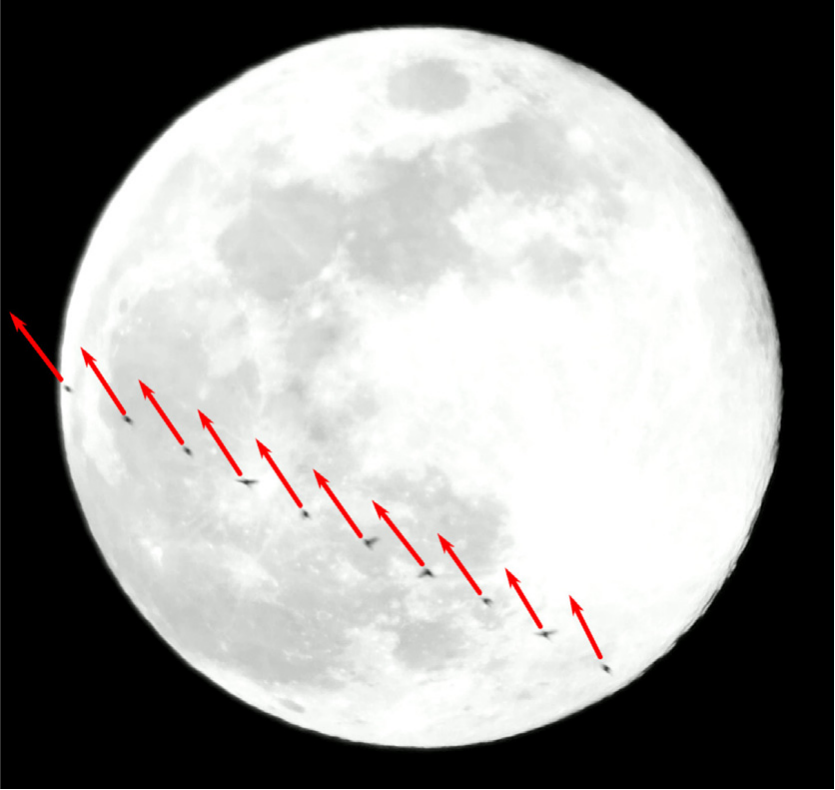
Composite image of a migrant crossing in front of the moon over the course of 10 frames recorded on April 28^th^, 2018. Arrows show the orientation of the bird’s body, as reckoned by drawing a line from the tail to the beak.

We have demonstrated that video analysis can be used to derive precise flight directions for individual birds as well as the orientation of a bird’s body during flight (Fig. 6). Combined, these flight direction and orientation measures can be used to study wind drift and compensation behavior in migrating birds. Recent analyses of weather radar data indicate that it possible to estimate the aggregate body orientation of birds in flight and infer the degrees to which birds drift with the wind or compensate behaviorally [29]. Hence, LunAero offers an opportunity to validate these interpretations of weather radar data and help us better understand the behavioral strategies employed by migrating birds.

A few assumptions must be made to extract information about the flying birds. The accepted approach assumes that all observed migrants are passerines between 10 g–100 g, an approach gradually codified based on original quantification of nocturnal migrants by [20]. We calculate distance from the observer to the bird based on apparent size, which is simplified by counting the pixels of the shape of a silhouette. Estimates of distance are important in quantifying migration because with moon watching, the “cone” of observation widens as the distance from the observer increases. In other words, there is a much larger catchment volume for birds that are farther away, and a true density estimate must account for this factor. With rough estimates of distances available based on scope resolving power, we can offer more informative quantification of nocturnal migration than Lowrey’s traffic rate estimates [9], [30].

At present, the most commonly used methods for quantifying nocturnal migration involve some form of radar. Radar provides migration data on a variety of scales ranging from the continental data provided by the NEXRAD network of weather surveillance radars, to short-range marine radars and tracking radars capable of detecting individual birds. NEXRAD generates aggregate, macro-scale data based on reflected photons that can be attributed to a group of dozens to thousands of birds within a given radar volume. Estimating numbers of birds in a radar volume requires assumptions about the radar reflectivity of bird aggregations, and these assumptions have not been fully validated [31]. Moreover, weather radars do not provide information on the behaviors of individuals. In contrast, moon watching provides data which can be extrapolated to meso-scale, and it therefore has great potential to compliment and inform the macro-scale data available from the NEXRAD network. Moreover, we hope that as more data accumulate, moon watching can provide the ground-truthing necessary to calibrate estimates of bird densities based on radar reflectivity.

Moon watching has several inherent limitations, even when enhanced with LunAero.

• **Moon watching relies on moon phase.** Although we have not fully tested the limits of LunAero and the moon watching method, we think that it would only be effective even when the moon is at least >50% full.
• **Moon watching necessitates clear skies.** Even a brief obfuscation of the moon by clouds can cause LunAero to lose its fix on the moon’s position. At present we have no means for LunAero to recover from having lost the moon, although it would be possible to implement a search pattern.
• **Moon watching samples a small portion of the sky.** If factors such as artificial light, landscape features, or wind phenomena affect the specific routes taken by nocturnal migrants, then there is the potential for biased migration estimates depending on the particular location of moon watching equipment relative to these factors.

Additionally, quantifying the birds recorded in LunAero video samples is time-intensive, taking at least ten times as long as real-time observation. We regard this time expenditure as a trade-off rather than a distinct limitation, attempting to quantify LunAero recordings produces more accurate results at the cost of time investment.

By applying modern computing to nocturnal migration bird watching, we have advanced moon watching by not only making it more convenient, but by improving it’s accuracy and utility as a research method. Not counting the cost of a spotting scope, a LunAero system can be assembled for <$400. By keeping the cost low we ensure the practice of moon watching remains accessible and within the means of a citizen scientist with a strong interest in migration. Although moon watching has not been as widely employed as other research techniques since Lowery’s efforts in the 1950s and 1960s, we argue that our automated recording system may help this classic technique find a niche in modern migration studies.

## Declaration of interest

The authors would like to acknowledge funding from the University of Oklahoma’s Strategic Organization in Applied Aeroecology and donors from the LunAero crowdfunding campaign. Declarations of interest: none.

## Human and animal rights

The work presented in this manuscript did not involve handling or materially affecting animals. As such, it was exempted from review by the University of Oklahoma Institutional Animal Care and Use Committee.
